# Statistical analysis plan for the LAKANA trial: a cluster-randomized, placebo-controlled, double-blinded, parallel group, three-arm clinical trial testing the effects of mass drug administration of azithromycin on mortality and other outcomes among 1–11-month-old infants in Mali

**DOI:** 10.1186/s13063-023-07771-6

**Published:** 2023-11-15

**Authors:** Juho Luoma, Laura Adubra, Dagmar Alber, Per Ashorn, Ulla Ashorn, Elaine Cloutman-Green, Fatoumata Diallo, Camilla Ducker, Riku Elovainio, Yue-Mei Fan, Lily Gates, Gwydion Gruffudd, Tiia Haapaniemi, Fadima Haidara, Lotta Hallamaa, Rikhard Ihamuotila, Nigel Klein, Owen Martell, Samba Sow, Taru Vehmasto, Yin Bun Cheung

**Affiliations:** 1 Center for Child, Adolescent and Maternal Health Research, Faculty of Medicine and Health Technology, Tampere University, Tampere, Finland; 2 Great Ormond Street Institute of Child Health, University College London, London, UK; 3 Department of Paediatrics, Tampere University Hospital, Tampere, Finland; 4 Center for Vaccine Development, Bamako, Mali; 5 Tro Da Ltd, Cardiff, UK; 6 Program in Health Services and Systems Research and Centre for Quantitative Medicine, Duke-NUS Medical School, Singapore, Singapore

**Keywords:** Cluster randomized trial, Statistical analysis plan, Antibiotic, Azithromycin, Mortality, Morbidity, Growth, Inflammation, Antimicrobial resistance

## Abstract

**Background:**

The Large-scale Assessment of the Key health-promoting Activities of two New mass drug administration regimens with Azithromycin (LAKANA) trial in Mali aims to evaluate the efficacy and safety of azithromycin (AZI) mass drug administration (MDA) to 1–11-month-old infants as well as the impact of the intervention on antimicrobial resistance (AMR) and mechanisms of action of azithromycin. To improve the transparency and quality of this clinical trial, we prepared this statistical analysis plan (SAP).

**Methods/design:**

LAKANA is a cluster randomized trial that aims to address the mortality and health impacts of biannual and quarterly AZI MDA. AZI is given to 1–11-month-old infants in a high-mortality setting where a seasonal malaria chemoprevention (SMC) program is in place. The participating villages are randomly assigned to placebo (control), two-dose AZI (biannual azithromycin-MDA), and four-dose AZI (quarterly azithromycin-MDA) in a 3:4:2 ratio. The primary outcome of the study is mortality among the intention-to-treat population of 1–11-month-old infants. We will evaluate relative risk reduction between the study arms using a mixed-effects Poisson model with random intercepts for villages, using log link function with person-years as an offset variable. We will model outcomes related to secondary objectives of the study using generalized linear models with considerations on clustering.

**Conclusion:**

The SAP written prior to data collection completion will help avoid reporting bias and data-driven analysis for the primary and secondary aims of the trial. If there are deviations from the analysis methods described here, they will be described and justified in the publications of the trial results.

**Trial registration:**

ClinicalTrials.gov ID NCT04424511. Registered on 11 June 2020.

## Background

Mass drug administration (MDA) of azithromycin (AZI) is a promising strategy for improving child survival in settings with high under five mortality rates (U5MR) [1]. According to an expert insight, the positive impact of AZI MDA is likely mediated through azithromycin’s activity against bacterial pathogens of the lung and gastrointestinal tract, or the plasmodial apicoplast, that in turn could lead to reduced incidences of respiratory infections, diarrhea, and malaria [1]. The available evidence is, however, limited, and the World Health Organization (WHO) has called for further research including that on optimal dose, frequency and number of intervention cycles, and potential harms, notably antimicrobial resistance (AMR).

LAKANA stands for Large-scale Assessment of the Key health-promoting Activities of two New mass drug administration regimens with Azithromycin. The primary aim of this trial conducted in Mali, covering 1151 villages, is to evaluate the impact on mortality of two or four annual rounds of AZI MDA delivered to 1–11-month-old infants. The study also includes a range of secondary outcomes such as efficacy outcomes related to morbidity and growth, outcomes related to the mechanism of azithromycin activity through measures of markers of infection and inflammation, safety outcomes (AMR, adverse, and serious adverse events), and outcomes related to the implementation of the intervention documenting feasibility, acceptability, and economic aspects. The LAKANA trial has been registered with ClinicalTrials.gov (NCT04424511) and its protocol (version 4.0, dated 27 June 2022) has been published [2]. In the current paper, we describe the statistical analysis plan (SAP) that has been finalized prior to the last follow-up visit before interim analysis of the last enrolled participants and to which the trial data analysis will adhere. Published guidance on the contents of SAPs was used as reference to cover essential items [3].

## Trial design

The study is a three-arm cluster randomized, placebo-controlled, double-blinded, parallel-group clinical trial. The trial was designed to estimate the impact of azithromycin on 1–11-month-old infant mortality in Mali, Western Africa, with a sample size of 1151 villages (clusters). The area is covered by a national seasonal malaria chemoprevention (SMC) program that was designed to include distribution of sulfadoxine-pyrimethamine plus amodiaquine to all l–59-month-old-children monthly during the rainy season (July to October). Details of the trial design are described in a separate protocol article [2].

Participating study villages were randomly allocated into the three intervention groups at 3:4:2 ratio: placebo (control), two-dose AZI (azithromycin-MDA treatments given every 3 months to 1–11-month-old infants between January and June), and four-dose AZI (azithromycin-MDA treatments given every 3 months to 1–11-month-old infants). The randomization was stratified by village size: villages for which the estimated number of eligible infants was 100 or more (later referred to as “big villages”) or villages for which the estimated number of eligible infants was fewer than 100 (later referred as “small village”). The village size categorization was done based on 2018 Demographic and Health Survey [4]. The unit of allocation is village, unit of treatment with a study drug is 1–11-month-old infant, and unit of enrolment is a household representative.

The data collection team visits the villages at quarterly intervals, eight times for MDA visits and one time for close-out visit (visit 9) during which the data collection team interviews the household representatives but do not administer study drug. A single child can receive up to four doses of study drug between the age range of 1 and 11 months, even though the villages are visited eight times at maximum. The trial may have equal stopping time for all of the villages, despite staggered starting time, if the target person-years-at risk will be reached before all the villages have had full visit cycle of nine visits. If the target person-years-at-risk requires full visits for all of the villages, there will be nine visits for all villages. The decision will be made by the Project Steering Group (PSG) after the interim analysis based on the recommendation by the Data Safety and Monitoring Board (DSMB). At minimum, all villages will be visited six times.

In a subset sample of 59 villages, the study team is collecting additional biological samples and measurements to address secondary study questions about AMR, growth and nutritional status, and mechanisms of action of azithromycin. The 59 villages were chosen based on their proximity to health centers in Kita area, as well as their location in clearly rural area. The selection was based both on the possibility to deliver biological samples to a laboratory in reasonable time, as well as being rural and therefore representative of most of Mali.

The trial DSMB will conduct a single interim analysis when approximately 60% of the mortality data has been collected, and based on the results of the interim analysis, a trial arm may be dropped, or the trial may be stopped early [2].

### Current status of trial

Enrollment for LAKANA began on October 15, 2020, and ended in December 2022. Data collection is ongoing and the last follow-up for the final participants is currently planned to take place in the second half of 2024. We estimate the interim analysis to take place in August 2023.

## Study objectives

### Main objective

The main objective of this trial is to determine the impact of a public health intervention program of two-dose (given twice a year, between January and June) or four-dose (given every 3 months) AZI MDA to 1–11-month-old infants on their mortality, when provided in a context of a national SMC program.

### Secondary objectives

In addition to the primary aim, the trial has secondary aims in assessing other health effects of AZI on the treated population. The secondary aims are as follows:

i) To evaluate the effect of receiving two-dose and four-dose AZI MDA on AMR.
ii) To evaluate the impact of AZI MDA to 1–11-month-old infants on their period prevalence of acute infection symptoms.
iii) To evaluate the impact of AZI MDA to 1–11-month-old infant on their linear growth, weight gain, and prevalence of malnutrition.
iv) To investigate mechanisms of AZI action by testing hypotheses on
  whether or not AZI MDA eliminates malaria parasitaemia.
  whether or not AZI MDA increases mean blood hemoglobin concentration.
  whether or not AZI MDA reduces systemic or intestinal inflammation in asymptomatic children.

Also, an integrated LAKANA feasibility study aims to provide comprehensive policy advice to decision makers on possible strategies to implement an AZI intervention as a national program should the trial yield positive clinical results. Feasibility study is not described in this SAP due to many aspects of the study being conditional on the trial results.

## Outcomes

### Primary outcome

The primary outcome of the trial will be 1–14-month-old mortality following the 3-month interval after the village has received the AZI MDA or placebo. We record the vital status of village residents as categorical variable with four options: “alive”, “dead”, “moved”, and “unknown”. In the mortality analysis, the numerator will be binary (alive/dead). For participants whose information on vital status at the end of a 3-month interval, we cannot obtain or infer based on subsequent visits, the interval will be excluded. The denominator, follow-up time, is calculated by subtracting date of first treatment from date of the last vital status or date of death, whichever is earlier. The obtained difference in days is divided by 365.25 to obtain person-years-at-risk (PYR). If the date of death is not obtained for any reason, we will use the mid-point between previous visit and date when death is reported as an approximation.

The treatment variable is a three-level categorical variable:

- Control: category for infants in villages that receive placebo at every visit
- Two-dose AZI: category for infants in villages that receive azithromycin two times in a year
- Four-dose AZI: category for infants in villages that receive azithromycin at every visit

### Secondary outcomes

#### Antimicrobial resistance

We will express AMR as the proportion of *E. coli* or *S. pneumoniae* isolates that have reduced susceptibility, as in “resistant” (R) or “intermediate” (I) category, primarily towards azithromycin and secondarily towards a subset of other antibiotics that may have overlapping mechanisms: meropenem, ceftriaxone, ciprofloxacin, ampicillin, co-trimoxazole, and gentamicin for *E. coli* and oxacillin, erythromycin, vancomycin, ciprofloxacin, ampicillin, and co-trimoxazole for *S. pneumoniae*.

The nasopharyngeal and rectal samples will be collected from 4–14-month-old and 49–59-month-old children at the first visit to the village, at 12 months (MDA 5), at 24 months (visit 9), and at 36 months, i.e., 1 year after the last MDA for the villages. We will primarily calculate the proportion of R/I isolates from all isolates and secondarily the proportion of children with R/I isolate in their sample.

We will test a sample of 1350 children aged 4–14 months at 24 months and aged 49–59 months at 36 months. At the three other time points, we will test 450 children (taken randomly from approximately 20 clusters/arm) from 4–14 months old and 49–59 months old, respectively. In total, this will mean 5400 AMR-analyses for *S. pneumoniae* and the same number for *E. coli*.

**Fig. 1:**
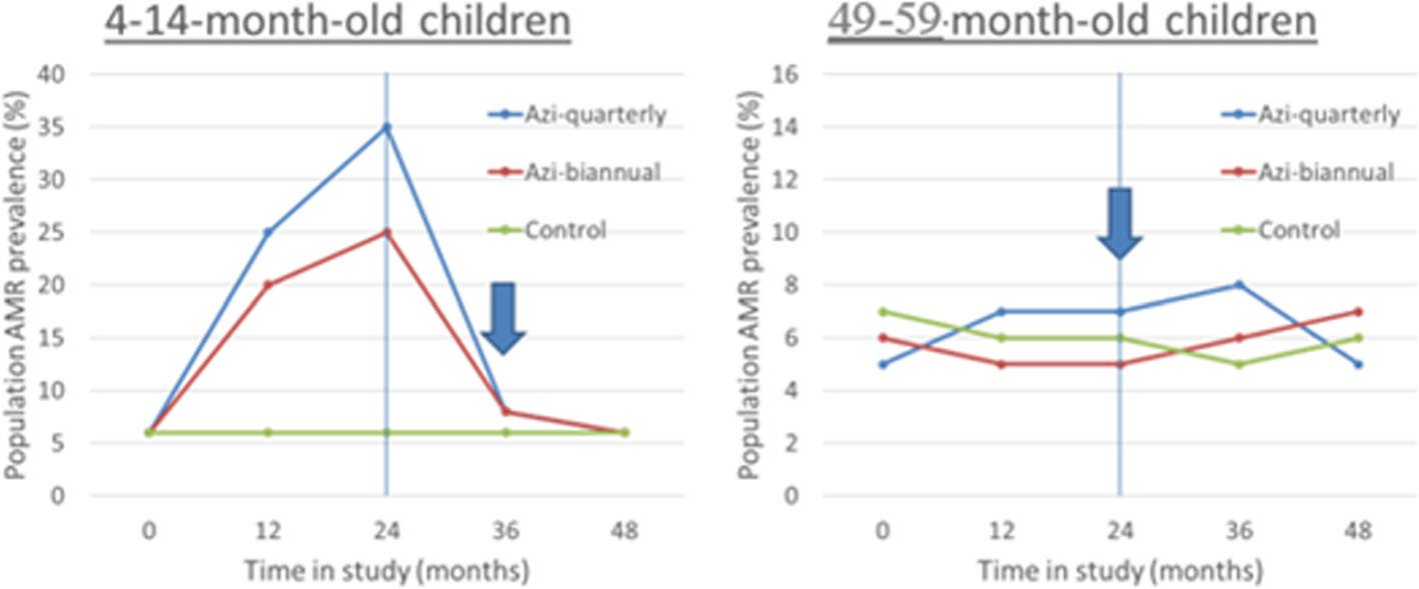
The assumptions of AMR prevalence at baseline and follow-up visits in the 4–14-month-old and 49–59-month-old children. Graph is generated with hypothetical data for illustration of what the study team hypothesizes

We hypothesize the prevalence of AMR will exhibit an initial increase among 4–14-month-old individuals, followed by a subsequent decrease after cessation of the MDA intervention. In comparison, we anticipate that AMR prevalence will remain stationary among 49–59-month-old individuals across all three study arms throughout the course of the MDA cycles, as well as following the cessation of the intervention (Fig. 1).

We will employ probability weighting to account for the stratified sampling procedure used to select individuals from the village-age strata. The weights are calculated as the inverse of the selection probability for each individual in the sample, which enables weighted analysis of the dataset.

#### Growth and nutritional status

Length-for-age Z-score (LAZ), weight-for-age Z-score (WAZ), weight-for-length Z-score (WLZ), and mid-upper arm circumference Z-score (MUAC-Z) will be calculated using the WHO Child Growth Standards [5]. We will calculate the prevalence of moderate-to-severe and severe stunting (LAZ <  − 2 / LAZ <  − 3) and moderate-to-severe and severe wasting (WLZ <  − 2 / WLZ <  − 3) as percentages. Data collection team will record growth and nutrition status information from 6–8-month-old and 12–14-month-old children at 15, 18, 21, and 24 months after village enrolment (MDAs 6–8 and visit 9).

#### Morbidity

Fourteen-day period prevalence of fever with acute respiratory infection (ARI), fever without respiratory symptoms (potential sign of malaria), and diarrhea prior to the visit in children aged 4–14 months will be assessed in each participating cluster (village) from the 59-village subset on each MDA.

#### Mechanisms of azithromycin action

The study nurses collect biological samples (blood, dry blood spot, and stool) for the secondary aims related to azithromycin action mechanisms twice: once before drug administration and 14 days after the drug administration. Biological sample collection takes place at the fourth or fifth MDA round (i.e., 9 months after village enrollment). The outcomes for the mechanistic objectives are as follows:

- *Parasite density.* Parasite density will be estimated from blood samples with polymerase chain reaction (PCR) cycle threshold (Ct) values. Ct values are plotted against the logarithmic concentrations of 3D7 control gDNA and sample concentrations are calculated (µg/mL). We use the attained sample concentration variable as a proxy for parasite density as previous studies have shown significant correlation between microscopy-defined parasite densities and PCR-defined Ct values [6].
- *Prevalence of malaria infection.* The laboratory team will assess malaria parasitemia prevalence with PCR from a blood samples. Ct value of less than or exactly 38 will be used to indicate positive samples.
- *Blood hemoglobin concentration (g/dL).* As a marker of health consequences of malaria treatment, the nurses measure blood hemoglobin concentration.
- *General inflammation.* C-reactive protein (CRP)-concentration (μg/mL) will be measured from blood samples to represent general inflammation.
- *Intestinal inflammation.* Alpha-1-antitrypsin (AAT) (mg/g), neopterin (NEO) (nmol/L), and myeloperoxidase (MPO) (ng/mL) concentrations will be measured from stool samples and combined into an environmental enteric dysfunction marker (EE score) [7]. We will also conduct statistical analyses on the inflammation markers individually.

#### Safety outcomes

*Incidence of serious adverse events (SAE).* We will collect information on incidence of SAEs within 14 days of study drug administration throughout the study.

*Incidence of adverse events (AE).* We will collect information on incidence of AEs within 14 days of study drug administration from 4–14-months-old infants from the 59-village subset at MDA 4 (i.e., 9 months after enrolment).

## Hypotheses

### Mortality effect

The hypotheses in terms of mortality effect are as follows:

i) There are fewer deaths per 1000 PYRs among 4–14-month-old infants in clusters where 1–11-month-old-infants are treated with two-dose AZI MDA than in villages where 1–11-month infants are treated with respective placebo.
ii) There are fewer deaths per 1000 PYRs among 4–14-month-old infants in clusters where 1–11-month-old-infants are treated with four-dose AZI MDA than in villages where 1–11-month infants are treated with respective placebo.
iii) There are fewer deaths per 1000 PYRs among 4–14-month-old infants in clusters where 1–11-month-old-infants are treated with four-dose AZI MDA than in villages where 1–11-month infants are treated with two-dose AZI MDA.

In addition to the aforementioned, we will conduct exploratory analyses to assess mortality among children who were 12–59 months old when the latest AZI MDA took place in their village of residence and who had not been eligible to receive azithromycin on previous MDA visits.

For hypothesis generating purposes, we will carry out exploratory analysis to investigate whether the following selected variables modified the effect of the MDA intervention on the mortality and growth outcomes:

- ◦ Age at the time of MDA
- ◦ Sex of the child
- ◦ LAZ
- ◦ WAZ
- ◦ WLZ
- ◦ Seasonality: rainy season vs non-rainy season
- ◦ SMC given in village
- ◦ Cluster level coverage of SMC (in %)
- ◦ Cluster level baseline mortality (established at MDA 1)
- ◦ Cluster level coverage of AZI MDA
- ◦ Individual level coverage and the number of administered AZI doses
- ◦ District of residence
- ◦ Distance from the nearest health facility (in km)
- ◦ Household asset index
- ◦ Water, sanitation, and hygiene (WASH) index

The household asset index and WASH index will be developed by principal component analysis. The component that explains the largest proportion of variance from household assets and WASH questionnaires will be used as the indices [8].

### AMR

To evaluate the effect of different MDA frequencies on AMR, we will test 12 hypotheses:

i) Among 4–14-month-old children, the prevalence of AZI-resistant *S. pneumoniae* or *E. coli* strains isolated at 12 months after cessation of the intervention in the village is not higher in villages that receive two-dose AZI regimen for 2 years than in villages that do not receive any AZI regimen during the same time frame.
ii) Among 4–14-month-old children, the prevalence of AZI-resistant *S. pneumoniae,* or *E. coli* strains isolated at 12 months after cessation of the intervention in the village is not higher in villages that receive four-dose AZI regimen for 2 years than in villages that do not receive any AZI regimen during the same time frame.
iii) Among 4–14-month-old children, the prevalence of AZI-resistant *S. pneumoniae,* or *E. coli* strains isolated at 12 months after cessation of the intervention in the village is not higher in villages that receive two-dose or four-dose AZI regimen for 2 years than in villages that do not receive any AZI regimen during the same time frame.
iv) Among 4–14-month-old children, the proportion of infants carrying AZI-resistant pneumococci in their nasopharynx or AZI-resistant *E. coli* in their stools at 12 months after cessation of the intervention in the village is not higher in villages that receive two-dose AZI regimen for 2 years than in villages that do not receive any AZI regimen during the same time frame.
v) Among 4–14-month-old children, the proportion of infants carrying azithromycin-resistant pneumococci in their nasopharynx or AZI-resistant *E. coli* in their stools at 12 months after cessation of the intervention in the village is not higher in villages that receive four-dose AZI regimen for 2 years than in villages that do not receive any AZI regimen during the same time frame.
vi) Among 4–14-month-old children, the proportion of infants carrying AZI-resistant pneumococci in their nasopharynx or AZI resistant *E. coli* in their stools at 12 months after cessation of the intervention in the village is not higher in villages that receive two- or four-dose AZI regimen for 2 years than in villages that do not receive any AZI regimen during the same time frame.
  We will also investigate older children (49–59 months old) for reference:
vii) Among children who are 49–59 month olds at the end of the trial, and who live in villages where MDA has been given to 1–11-month infants, but who have themselves not received any MDA, the proportion carrying AZI-resistant pneumococci in their nasopharynx after eight MDA rounds have been completed in the previous 2 years is not higher in villages that receive two- or four-dose AZI regimen than in villages that receive respective placebo regimen during the same time frame.

### Morbidity

The specific hypotheses regarding morbidity are the following:

i) Four to 14-month-old children in villages receiving two- or four-dose AZI regimen have lower prevalence of diarrhea within the last 14 days before MDA than same-aged children living in villages receiving placebo.
ii) Four to 14-month-old children in villages receiving two- or four-dose AZI regimen have lower prevalence of fever without cough within the last 14 days before MDA than same-aged children living in villages receiving placebo.
iii) Four to 14-month-old children in villages receiving two- or four-dose AZI regimen have lower prevalence of respiratory symptoms within the last 14 days before MDA than same-aged children living in villages receiving placebo.
iv) Children in villages receiving two- or four-dose AZI regimen have lower prevalence of any morbidity symptoms within the last 14 days before MDA than same-aged children living in villages receiving placebo.

### Growth and nutritional status

The specific hypotheses regarding growth and nutritional status are as follows:

i) Mean LAZ, WAZ, WLZ, and MUAC-Z measured at 15, 18, 21, and 24 months after village enrollment are higher among 6–8- and 12–14-month-old children in villages that receive two-dose AZI regimen than among same-aged children in villages that receive placebo.
ii) Mean LAZ, WAZ, WLZ, and MUAC-Z measured at 15, 18, 21, and 24 months after village enrollment are higher in 6–8- and 12–14-month-old infants in villages that receive four-dose AZI regimen than among same-aged children in villages that receive placebo.
iii) Proportion of children with LAZ, WAZ, WLZ, or MUAC-Z below − 2 at 15, 18, 21, and 24 months after village enrollment is higher in 6–8- and 12–14-month-old infants in villages that receive two-dose AZI regimen than among same-aged children in villages that receive placebo.
iv) Proportion of children with LAZ, WAZ, WLZ, or MUAC-Z below − 2 at 15, 18, 21, and 24 months after village enrollment is higher in 6–8- and 12–14-month-old infants in villages that receive four-dose AZI regimen than among same-aged children in villages that receive placebo.

### Mechanisms

As the investigation into the mechanisms of action of azithromycin centers on the dose-dependent impact at an individual level, specifically the underlying mechanisms of azithromycin’s function following administration, the two treatment arms may be consolidated for analysis. The mechanisms of azithromycin’s action shall be investigated through testing the following hypotheses:

i) Reduction in the prevalence of PCR-diagnosed malaria infections between day 0 and day 14 is greater in infants in villages receiving two-dose or four-dose AZI MDA regimen than in infants in villages receiving placebo MDA regimen.
  - Among those who test positive on day 0, the proportion of positive PCR test for malaria on day 14 is lower among infants who receive AZI MDA than among children who receive respective placebo.
ii) Reduction of intensity of subclinical malaria (expressed in parasite density) between day 0 and day 14 is greater in infants in villages receiving two-dose or four-dose AZI MDA regimen than in infants in villages receiving placebo MDA regimen.
iii) Increase in mean blood hemoglobin concentration between day 0 and day 14 is greater in infants in villages receiving two-dose or four-dose AZI MDA regimen than in infants in villages receiving placebo MDA regimen.
iv) Reduction in mean CRP concentration between day 0 and day 14 is greater in infants in villages receiving two-dose or four-dose AZI MDA regimen than in infants in villages receiving placebo MDA regimen.
  - Among infants with elevated CRP concentration (CRP > 5 μg/mL) at day 0, reduction in the prevalence of elevated CRP concentration is greater among infants who receive AZI MDA than among children who receive respective placebo.
v) Reduction in mean AAT, mean NEO, mean MPO concentrations, and EE score, between day 0 and day 14 is greater in infants in villages receiving two-dose or four-dose AZI MDA regimen than in infants in villages receiving placebo MDA regimen.
  - Among infants with elevated AAT/MPO/NEO concentration (AAT > 270 µg/g; MPO > 2000 ng/L; NEO > 70 nmol/L) at day 0, reduction in the prevalence of elevated AAT/MPO/NEO concentration is greater among infants who receive AZI MDA than among children who receive respective placebo.

## Statistical methods

### General principles

We will analyze the mortality outcome data by the intention-to-treat (ITT) principle, where the ITT population will include all infants of the eligible age at the time of the MDA visit from all the randomized villages according to the treatment the villages were randomized to receive. That is, if the household gives consent on participating to the study, the study team will offer to treat the child. Even if the child is not treated, their survival information will be analyzed. For the secondary outcome data from the subset of 59 villages, we will analyze the treated population, which only includes the infants who received at least one dose of the treatment drug.

Unless otherwise specified, we will use mixed-effects modeling approach to account for clustering, all the hypothesis testing will be at 5% two-sided significance level, and all confidence intervals will be 95% and two-sided, calculated by using robust standard errors. For calculating robust standard errors, we will use sandwich estimator of variance for clustered data. For the primary aim of the estimation of the effect of azithromycin treatment on 1–11-month-old mortality and for the secondary aims, we will carry out adjustment for multiple comparisons between groups by closed-testing procedure [9].

We conduct blind-review on the data during data collection and prior to any formal analysis. That is, we conduct exploratory analysis to check for errors and inconsistencies, without knowing the intervention group identities. Based on the blind-review of the data as well as previous literature, we will apply natural log transformation on the following biomarker variables: CRP, AAT, NEO, MPO, and parasite density [10]. We will not apply data transformations on hemoglobin variable as the blind review of the data suggests that the normality assumption is not violated.

### Analysis of the primary outcome

#### Azithromycin treatment effect on mortality among 1–11-month-old infants

We will tabulate the mortality (deaths/1000 PYR and absolute numbers) per arm and per 3-month intervals preceding the second to ninth visit. To investigate if azithromycin treatment reduces mortality among 1–11-month-old infants, we will estimate incidence rate ratio (IRR) and its 95% CI to compare the treatment regimens. We will use mixed-effects Poisson model to estimate intervention effects between treatment groups, with random intercepts for clusters (villages), using log link function with person-years as an offset variable. At maximum, an infant can receive the treatment at four MDAs, thus provide up to 1 year of follow-up time to the study data.

The main analysis hypothesis testing will be conducted as one-sided, that is, we hypothesize there to be lower mortality in the treatment groups. The decision to use one-sided test was based on the existing evidence of AZI having a potentially beneficial effect on early childhood mortality in previous trials [1] and our expectation is that we will recommend AZI if there is evidence of benefit, and we will not recommend AZI otherwise. This provides greater statistical power to detect differences between groups while being more appropriate for the study scenario than two-sided test.

In the main analyses, we will adjust for stratification factor in the randomization scheme (village size category) as a fixed effect. The main analysis will not make additional adjustments for covariates.

The mixed-effects model with log link function for the primary aim on intervention effect on mortality will provide the IRR. To understand the population importance of the intervention, we will use non-linear combinations of the parameter estimates from the same mixed-effects model to obtain average estimates of incidence rate difference (IRD) [11]. In this procedure, exponentiation of the estimate of intercept is the average mortality rate in the placebo group, whereas exponentiation of the sum of the estimates of intercept and two-dose (four-dose) coefficient is the average mortality rate in the two-dose (four-dose) groups. IRD can then be derived, with confidence interval obtained using the delta method [12].

### Analysis of the secondary outcomes

#### The effect of MDA regimes on AMR

We will present the proportions of children with resistant isolates in at baseline (i.e., MDA 1) and at visit 9 for all the trial arms. We will also produce a table with proportions of resistant isolates from all isolates in the same manner as for children with resistant isolates. To estimate the impact of two- or four-dose AZI regimen on the prevalence of phenotypic AZI resistance among *S. pneumoniae* or *E.coli* strains isolated from 4–14-month-old children, we will fit a binomial regression model with identity link to obtain the risk difference with 95% CI. We will use robust standard errors to account for clustering in the analysis of the effect of MDA regimes on AMR, since using mixed-effects modeling with link functions suitable for deriving risk difference, such as the identity or log-link, can pose conceptual challenges. Specifically, estimates derived from such models may not be valid due to the unbounded nature of the random intercept’s variance without additional constraints. This can result in estimated proportions falling outside the allowable range of 0 to 1.

If the entire 95% CI for the time point at 1 year after the last MDA in the treatment arms is below a predefined non-inferiority margin of + 10 percent points as compared to the control arm, the sample findings will be considered supportive of the hypothesis of no clinically significant increase in AMR prevalence. The procedure will be same for 49–59-month-old children.

#### The effect of MDA regimens on morbidity

For morbidity analyses, we will calculate the 14-day period prevalence of fever with ARI, fever without respiratory symptoms, and diarrhea in the three intervention arms and perform a three-group comparison using likelihood ratio tests. The latter will be obtained from mixed-effects logistic regression models, with random intercepts for clusters and children to allow within-cluster correlation in clusters and timepoints. If the global test of null hypothesis shows a statistically significant difference (*p* < 0.05) between the three groups, we will proceed into three pair-wise comparisons, providing relative risks (RR) and their 95% CIs by predicting the marginal risk ratios from the model coefficients [13]. If both the global and pairwise *p*-value are < 0.05, the results will be considered statistically significant.

#### The effect of MDA regimens on growth and nutritional status

We will tabulate the descriptive summaries (mean, SD, min, max) on LAZ, WAZ, WHZ, and MUAC-Z score for each treatment arm. We will analyze the impact of AZI MDA on growth and nutritional status using mixed-effects regression model with identity link and random intercepts for clusters and for children.

In addition, we will perform analyses on dichotomous outcome variables, including moderate-to-severe and severe stunting (LAZ <  − 2/LAZ <  − 3), moderate-to-severe and severe underweight (WAZ <  − 2/WAZ <  − 3), and moderate-to-severe and severe wasting (WLZ <  − 2/WLZ <  − 3). We will tabulate the proportions of stunted, underweight, and wasted per treatment arm and age group (6–8 months, 12–14 months, respectively) and calculate their differences (95% CI). We will test a hypothesis that there is no difference between the treatment arms with respect to the prevalence of stunting or wasting by using a mixed-effects logistic regression model with random intercepts for clusters and for children. The marginal risk ratios will be predicted from the obtained model coefficients.

#### The effect of MDA regimens on mechanisms of action of azithromycin

Outcomes related to study questions on mechanisms of action of azithromycin are measured twice: at day 0, i.e., before the treatment on the day of fourth MDA visit (9 months after village enrolment), and 14 days after. The two intervention groups will be combined in the analysis. For each endpoint in the mechanistic study, mixed-effects regression model with random intercepts for clusters will be estimated, including intervention (versus control) and the day 0 measure as the independent variables and the day 14 measure as the dependent variables.

To investigate the impact of AZI MDA on malaria parasitaemia prevalence, the prevalence of elevated CRP concentration (CRP > 5 µg/mL), prevalence of elevated AAT concentration (AAT > 270 µg/g), MPO concentration (MPO > 2000 ng/L), and NEO concentration (NEO > 70 nmol/L), we will use the binary variables as dependent outcome variables in mixed-effects logistic regression models with random intercepts for clusters and for children. The marginal risk ratios will be predicted from the obtained model coefficients. Risk ratios and their 95% CIs will be reported.

We will model the impact of AZI MDA on the intensity of subclinical malaria by using the natural logarithm transformed values from the parasite density variable as the dependent outcome variable in a mixed-effects model with random intercepts for clustering and identity link function. We will use the same modeling approach on modeling the impact of AZI MDA on general inflammation and intestinal inflammation (that is, CRP concentration, AAT concentration, MPO concentration, and NEO concentration, respectively). We will report the exponentiated value of the regression coefficient (geometric mean) and their 95% CI. We will conduct the same analysis with the blood hemoglobin concentration as the dependent outcome variable but report regression coefficients and their 95% CIs without exponentiation.

We will also produce tabulations on the descriptive summaries (means, medians, standard deviations, minimum, maximum) of the variables and report proportions and absolute numbers on positive and negative malaria parasitemia prevalences, as well as proportions and absolute numbers on elevated CRP/AAT/MPO/NEO concentration levels. The tabulations will be done respectively for day 0 and day 14.

### Subgroup analyses

To examine the effect modification, we will construct different models to test for interaction with pre-defined set of baseline variables. Hypothesis test of difference between groups within each stratum will be performed only if the interaction test gives statistically significant results (*p* < 0.1).

### Loss to follow-up and missing data

**Fig. 2:**
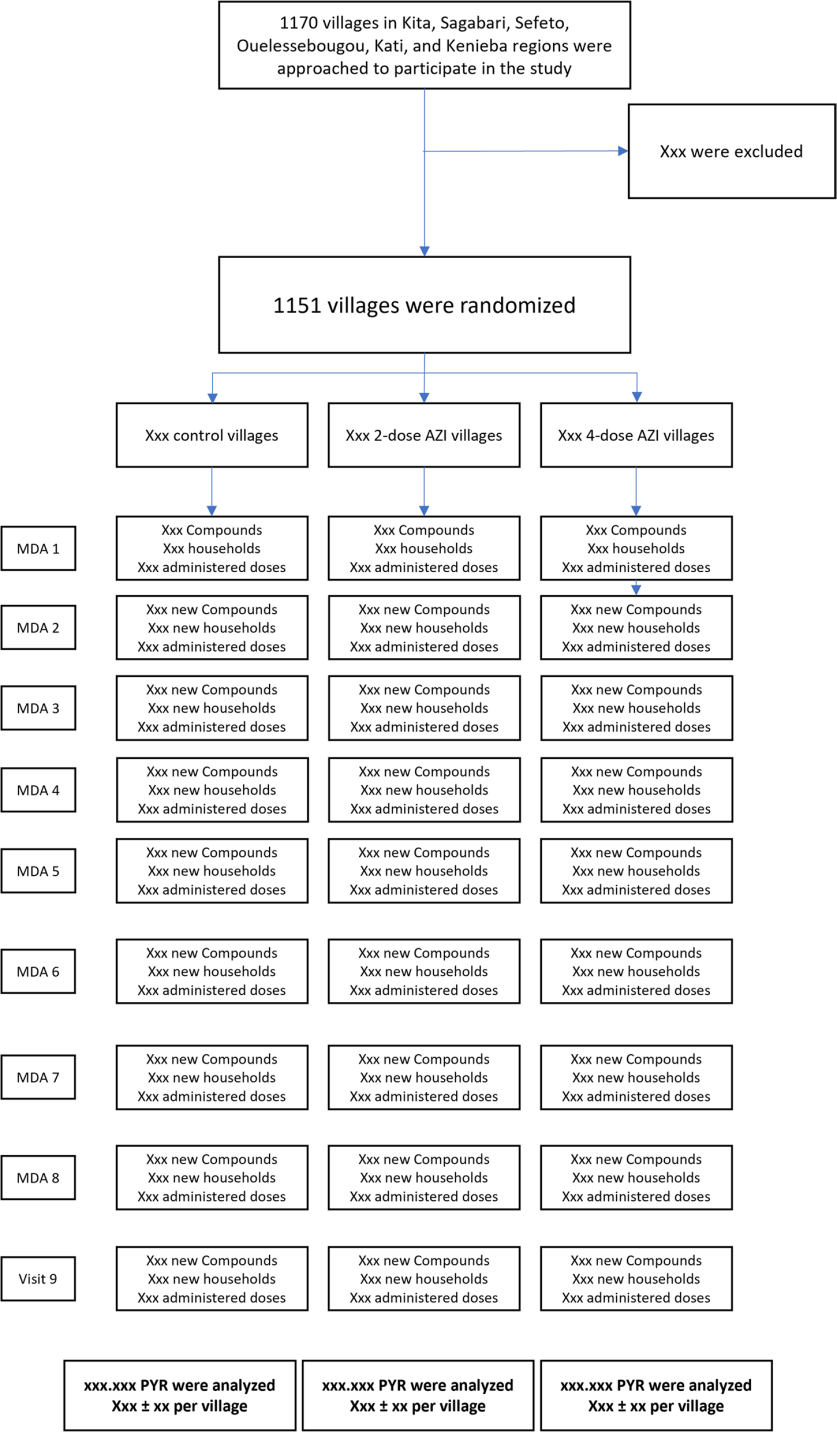
Trial profile

A participant will be deemed lost to follow-up, if vital status cannot be ascertained in any of the subsequent MDAs. We will present the numbers of loss to follow-up between the three trial arms in the trial flow diagram (Fig. 2). If a child is missing a visit at any given time point, but the vital status and visit date can be ascertained based on the information of the next visit, a visit date will be imputed based on the visit dates of other households within the same village. If date of death is missing from the analysis, we will use the mid-point between the last available visit date when the infant was alive and the latest visit date when the infant was known to have died as an approximation.

### Interim analysis

An interim analysis will be conducted when approximately 60% of the target PYR is available. This interim analysis will focus on mortality and SAEs. For this analysis, statistical significance is defined as two-sided *p*-value < 0.001 as per the Peto’s rule [9]. The analytic codes will be prepared by the study team and provided to the DSMB statistician along with study data required to run the analytic codes. The drug codes will be provided to the DSMB by a party not involved in trial implementation, in a format the analytical script can automatically merge into the data set for the analysis. The data and the analytical script will be password encrypted, and the password for data access will be provided to the DSMB statistician via separate, encrypted file sender. At the time of the interim analysis, only the DSMB statistician will be able to merge the actual drug codes to the study data, break the blinding, and conduct the analysis. The study data provided to the DSMB statistician will be de-identified prior to data transfer. The rationale in requesting a non-research team party to carry out the interim analysis is to keep the study data management blinded to the trial actual drug letter codes until the end of the trial.

If the interim analysis reveals statistically significant mortality difference between the quarterly azithromycin-MDA group and the placebo group and between the biannual azithromycin-MDA group and the placebo group but not between the two azithromycin groups, the placebo arm will be discontinued, and the trial will continue with two arms only. In this scenario, the villages with remaining MDA rounds in the placebo arm will be re-randomized to either of the treatment arms. If there is evidence of a mortality benefit in one or both azithromycin groups and also a statistically significant difference between the two azithromycin groups, the trial will be stopped, and the team will offer to work with Malian Ministry of Health to provide AZI MDA in the trial site and elsewhere in the regions of Mali, choosing the MDA regimen (quarterly or biannual) based on the trial data [3].

### Safety analysis

Written reports of any SAEs will be prepared during the duration of the trial and AEs will be reported as part of the data collection. Types, severity, and relatedness to intervention of the SAEs will be reported. We will not conduct formal group comparisons or statistical inference for the SAEs.

### Sample size considerations

#### Power-by-simulation approach

Simulations were conducted to determine the appropriate sample size to achieve target level of power for the main objectives as well as to confirm that the procedures related to the interim analysis will not compromise type I error rate [14]. The simulation assumptions are as follows:

1. Mortality among 1–11-month-(29–364 days)-old children in control group of 20 deaths/1000 PYR.
  - Justification for the baseline mortality estimate and respective assumptions on reduction in mortality had been described in separate protocol article [2].
2. Mortality in the two-dose AZI intervention group 16/1000 (20% relative reduction)
3. Mortality in the four-dose AZI intervention group 12/1000 (40% lower than control, 25% lower than two-dose AZI)
4. Two-year intervention with eight quarterly cycles of MDA for 50% of clusters, seven rounds for 30%, and six rounds for 20%, due to staggered entry but equal stopping calendar date.
5. Coefficient of variation (k = sd/mean) (CV) of 0.1 in mortality among clusters.
  - According to our estimate of 20 deaths/1000 PYR, the intra-cluster correlation (ICC) is estimated to be 0.0001 according to the findings of Gulliford et al. [15] on the relationship between ICC and event frequency. Plugging this ICC estimate to the formula developed by Thomson et al. [16] to convert ICC to CV, giving a CV of 0.07, which we rounded to 0.1 to be conservative.
6. Unequal number of infants per cluster: on average, 22 infants in a small village and 70 in a big village. Average for number of infants in a village being 31.
  - Average number of infants in small and big villages (respectively) was simulated with Poisson process. Based on prior information from local census data, we inferred the mean size to be 22 for small villages and 70 for big villages.
  - From the same data, we inferred the proportions for big and small villages to be 18% and 82%, respectively.
7. Sample size of 1150 villages
8. Unequal ratio of clusters. Control vs AZI-biannual vs AZI-quarterly = 3: 4: 2
9. One interim analysis when 60% of the planned 3-month time intervals have been completed.
10. One-sided 2.5% type 1 error, controlling of multiple pairwise comparisons by the closed-testing procedure
  - Closed-testing procedure will be carried out by calculating a *p*-value for the global null hypothesis of the regression coefficients being equal to zero (using Wald’s test). Pairwise null hypotheses can be rejected only if the two-sided *p*-value for the global null hypothesis is lower than 0.05
11. As per the Peto’s rule, significance level is set to 0.001 at interim analysis leading to either continuing the study to second phase, dropping the control arm, or stopping the trial early.

Simulations were run with 2000 iterations using aforementioned values for parameters. The simulations show that by specifying the aforementioned inputs, the study has approximately 89% power for testing the hypothesis that biannual AZI MDA will reduce mortality, > 99% power for testing the hypothesis that quarterly AZI MDA will reduce mortality, and 80% power for testing the hypothesis that quarterly AZI MDA will reduce mortality more than biannual AZI MDA. The simulations also showed that with the chosen analytical approach familywise type I error rate is controlled at the target level despite multiple pairwise comparison and interim analysis.

#### AMR

The following assumptions were used for the AMR sub-sample size calculations:

The AMR sub-sample size is based on the following assumptions.

1. 95% *E. coli* and 65% *S. pneumoniae* recovery rate from the collected samples
2. AMR prevalence of 12% in the control group for *E. coli* and 6% for *S. pneumoniae*. These figures come from analysis of azithromycin resistance in 50 *E. coli* and 50 *S. pneumoniae* samples from Mali in the so called ABCD trial.
3. Non-inferiority margin of 10 percent-points in AMR prevalence.
4. 80% power, one-sided 2.5% type 1 error rate for each pairwise comparison against placebo control.
5. Coefficient of variation of 0.3 in AMR among clusters.

### Statistical software

The analyses will be conducted using Stata statistical software. R programming language will be used for data pre-processing and other analyses if necessary. The used packages and version numbers will be included in the final report.

#### Data management

Data management is described in detail in separate standardized operating procedure (SOP) document as mentioned in the protocol article [2].

## Conclusion

LAKANA will provide valuable information on effect of different MDA strategies on 1–11-month-old mortality and health outcomes in Mali. This article provides details of the statistical analysis strategies planned for the trial data, aiming to reduce the risk of data driven results, as well as reporting bias. Large cluster randomized study design aims to evaluate the effect of azithromycin treatment on infant mortality by adding in dimensions that have not been addressed in previous studies. Cluster randomized design also simulates how the potential intervention rollout could be applied, should there be a nationwide implementation program.

## Data Availability

Not applicable. Upon completion of the trial, de-identified data will be made publicly available per the funder, the Bill and Melinda Gates Foundation, policy.

## Abbreviations

AAT: Alpha-1-antitrypsin
AE: Adverse events
AMR: Antimicrobial resistance
ARI: Acute respiratory infections
AZI: Azithromycin
CRP: C-reactive protein
CSCom: Centre de Santé Communautaire
CV: Coefficient of variation
CVD-Mali: Center for Vaccine development-Mali
DHS: Demographic and Health Survey
DSMB: Data Safety and Monitoring Board
IRD: Incidence rate difference
IRR: Incidence rate ratio
ITT: Intention-to-treat
LAKANA: Large-scale Assessment of the Key health-promoting Activities of two New mass drug administration regimens with Azithromycin
LAZ: Length-for-age Z-score
MDA: Mass drug administration
MPO: Myeloperoxidase
MUAC: Mid-upper arm circumference
NEO: Neopterin
PYR: Person-years at risk
RR: Relative risks
SAE: Serious adverse events
SAP: Statistical analysis plan
SMC: Seasonal malaria chemoprevention
SOP: Standard operating procedure
U5MR: Under-5 mortality rate
WASH: Water, sanitation, and hygiene
WAZ: Weight-for-age Z-score
WLZ: Weight-for-length Z-score

## References

[1] Keenan JD, Bailey RL, West SK, Arzika AM, Hart J, Weaver J (2018). Azithromycin to reduce childhood mortality in sub-Saharan Africa. N Engl J Med.

[2] Adubra L, Alber D, Ashorn P, Ashorn U, Cheung YB, Cloutman-Green E (2023). Testing the effects of mass drug administration of azithromycin on mortality and other outcomes among 1–11-month-old infants in Mali (LAKANA): study protocol for a cluster-randomized, placebo-controlled, double-blinded, parallel-group, three-arm clinical trial. Trials.

[3] Gamble C, Krishan A, Stocken D, Lewis S, Juszczak E, Doré C (2017). Guidelines for the content of statistical analysis plans in clinical trials. JAMA.

[4] Institut National de la Statistique - INSTAT, Cellule de Planification et de Statistique Secteur Santé-Développement, ICF. Mali Demographic and Health Survey 2018. Bamako, Mali: INSTAT/CPS/SS-DS-PF and ICF; 2019. Available from: http://dhsprogram.com/pubs/pdf/FR358/FR358.pdf. Accessed 5 Dec 2022.

[5] Onis M, WHO Multicentre growth reference study group (2007). WHO Child Growth Standards based on length/height, weight and age: WHO Child Growth Standards. Acta Paediatr..

[6] Ballard E, Wang CYT, Hien TT, Tong NT, Marquart L, Pava Z (2019). A validation study of microscopy versus quantitative PCR for measuring Plasmodium falciparum parasitemia. Trop Med Health.

[7] Kosek M, Haque R, Lima A, Babji S, Shrestha S, Qureshi S (2013). Fecal markers of intestinal inflammation and permeability associated with the subsequent acquisition of linear growth deficits in infants. Am J Trop Med Hyg.

[8] Filmer D, Pritchett LH (2001). Estimating wealth effects without expenditure data-or tears: an application to educational enrollments in states of India. Demography.

[9] Marcus R, Eric P, Gabriel KR (1976). On closed testing procedures with special reference to ordered analysis of variance. Biometrika.

[10] McCormick BJJ, Lee GO, Seidman JC, Haque R, Mondal D, Quetz J (2017). Dynamics and trends in fecal biomarkers of gut function in children from 1–24 months in the MAL-ED study. Am J Trop Med Hyg.

[11] Norton EC, Miller MM, Kleinman LC (2013). Computing adjusted risk ratios and risk differences in Stata. Stata J Promot Commun Stat Stata.

[12] Oehlert GW (1992). A note on the delta method. Am Stat.

[13] Williams R (2012). Using the margins command to estimate and interpret adjusted predictions and marginal effects. Stata J Promot Commun Stat Stata.

[14] Feiveson AH (2002). Power by simulation. Stata J Promot Commun Stat Stata.

[15] Gulliford MC, Adams G, Ukoumunne OC, Latinovic R, Chinn S, Campbell MJ (2005). Intraclass correlation coefficient and outcome prevalence are associated in clustered binary data. J Clin Epidemiol.

[16] Thomson A, Hayes R, Cousens S (2009). Measures of between-cluster variability in cluster randomized trials with binary outcomes. Stat Med.

